# Discrepancies in the Detection of *PML::RARA* Gene Rearrangement by Fluorescent In Situ Hybridization Using Commonly Used Dual Color Dual Fusion Probes

**DOI:** 10.3390/diseases14010017

**Published:** 2026-01-02

**Authors:** Hanan S. Elsarraj, Karsten Evans, Sydney Graham, Shivani Golem

**Affiliations:** 1 Department of Pathology and Laboratory Medicine, Loyola University Medical Center, Maywood, IL 60153, USA; hanan.elsarraj@lumc.edu; 2 Department of Pathology and Laboratory Medicine, Kansas City Veterans Affairs Medical Center, Kansas City, MO 64128, USA; karsten.evans@va.gov; 3 Department of Pathology and Laboratory Medicine, University of Kansas Medical Center, Kansas City, KS 66160, USA; sgraham7@kumc.edu

**Keywords:** acute promyelocytic leukemia, *PML::RARA* fusion, FISH, qRT-PCR, insertional rearrangement, diagnostic limitations, disseminated intravascular coagulation

## Abstract

Background/Objectives: Acute promyelocytic leukemia (APL) is a medical emergency associated with life-threatening complications such as disseminated intravascular coagulation (DIC), necessitating prompt therapeutic intervention and rapid diagnostic confirmation. APL is characterized by a translocation of the *PML* gene (15q24) with the *RARA* gene (17q21), resulting in the *PML::RARA* fusion gene on the derivative chromosome 15. Atypical *PML::RARA* rearrangements may escape detection by standard FISH probes. This study highlights limitations of commonly used probe sets and underscores the need for alternative FISH probe sets and complementary molecular testing. Methods: Two unique APL cases with atypical *PML::RARA* rearrangements were identified in our laboratory. Each case was evaluated at diagnosis using two commercially available FISH probe sets from Abbott Molecular and Cytocell. Metaphase FISH was performed to characterize the atypical FISH signal pattern further, and qRT-PCR was used to confirm the presence of the *PML::RARA* transcript. Results: Both cases demonstrated atypical rearrangements with a single fusion signal. In the first case, the Abbott probe detected a single fusion signal, while the Cytocell probe was negative. Metaphase FISH revealed an insertion of the *PML* region near *RARA* on chromosome 17. In the second case, the Cytocell probe was positive, and the Abbott probe was negative; metaphase FISH demonstrated insertion of the *RARA* region near *PML* on chromosome 15. qRT-PCR confirmed the presence of the *PML::RARA* transcript in both cases. Conclusions: These findings reveal limitations in commonly used *PML::RARA* FISH probes and support reflex testing with alternative probes and molecular confirmation to ensure accurate diagnosis.

## 1. Introduction

Acute promyelocytic leukemia (APL) is a medical emergency that necessitates prompt therapeutic intervention to prevent life-threatening complications, most notably disseminated intravascular coagulation (DIC). Due to the urgency of treatment, a rapid diagnostic turnaround time (TAT) is essential. APL is characterized by a specific chromosomal translocation involving the *PML* gene located at 15q24 and the *RARA* gene at 17q21, resulting in the formation of a *PML::RARA* fusion gene on the derivative chromosome 15. Multiple breakpoints have been identified within both genes. The *PML* gene contains three well-defined breakpoint cluster regions (bcr) 1-3: bcr1 in intron 6, bcr2 in exon 6, and bcr3 in intron 3. Additionally, a rare bcr within the *PML* gene has been identified in intron 7, contributing to a less common variant of the *PML::RARA* fusion transcript. In contrast, the majority of *RARA* breakpoints are located within intron 2. These variations give rise to four distinct *PML::RARA* fusion transcripts [1].

While most *PML::RARA* fusions result from balanced translocations, a subset of cases arises from insertional events, where *PML* is inserted into *RARA* or vice versa. These atypical rearrangements are often referred to as “cryptic” or “masked” translocations because they can evade detection by conventional cytogenetic methods [2]. Fluorescence in situ hybridization (FISH) is a widely utilized diagnostic tool for APL, offering rapid TAT and high sensitivity for typical rearrangements. However, the design and genomic coverage of commercially available dual-color, dual-fusion *PML::RARA* FISH probe sets can impact their ability to detect rare, atypical rearrangements. This study presents two cases of APL with cryptic *PML::RARA* rearrangements, highlighting the limitations of standard FISH probes and emphasizing the importance of using alternative probe sets and complementary molecular testing for accurate diagnosis.

## 2. Materials and Methods

A retrospective review of newly diagnosed APL cases from 2020 and 2021 was performed. Eight cases were identified: four exhibited the typical *PML::RARA* rearrangement FISH signal pattern with two fusion signals, while the remaining four showed atypical patterns. Two of the atypical cases were excluded—one due to the absence of conventional karyotype results and the other because the atypical FISH pattern resulted from a three-way translocation. The remaining two atypical APL cases, suspected to involve cryptic insertion events of either *PML* or *RARA*, were further investigated. The institutional review board approved this review.

Diagnostic evaluation included morphological evaluation of blood or bone marrow, flow cytometry, conventional karyotyping, FISH, next-generation sequencing (NGS) using a 141-targeted myeloid gene panel, and quantitative real-time PCR (qRT-PCR) for *PML::RARA* transcript detection. Morphologic, flow cytometry, and qRT-PCR results were obtained as part of the patients’ clinical care and collected from the electronic health records.

**Figure 1:**
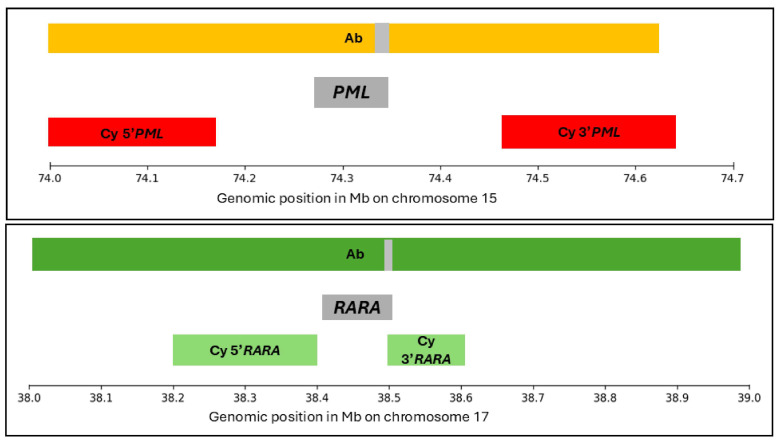
Genomic map of *PML* and *RARA* FISH probes from Abbott Molecular (Ab) and Cytocell (Cy). The *PML* probe coverage includes Ab orange probe spanning chr15:74,090,668–74,621,799 with a gap of chr15:74,329,383–74,343,964 (grey bar) and Cy Texas Red probes covering Cy 5′*PML* (chr15:74,026,937–74,179,715) and Cy 3′*PML* (chr15:74,468,806–74,644,928). The *RARA* probe coverage includes Ab green probe spanning chr17:38,090,884–38,926,745 with a gap of chr17:38,509,351–38,501,176 (grey bar) and Cy probes covering Cy 5′*RARA* (chr17:38,211,271–38,406,062) and Cy 3′*RARA* (chr17:38,501,211–38,667,419). Genomic coordinates are based on the human genome build GRCh37/hg19 and are presented in megabase pairs (Mb) [4,5].

Conventional cytogenetics was performed on the bone marrow aspirate culture, and analysis of 20 G-banded metaphases was performed using the Cytovision automated imaging system (Leica Biosystems, Nussloch, Germany). FISH was performed on the fixed cells using a dual-color dual-fusion *PML::RARA* probe set for the two cases investigated in this study. These FISH probes included LSI *PML*/*RARA* t(15;17) 05J70-001 *PML* orange and *RARA* green from Abbott (Ab) Molecular Inc, USA, and/or LPH501-A FAST *PML* in Texas red and LPH501-A FAST *RARA* in FITC from CytoCell (Cy) Ltd., Cambridge, UK [3], Figure 1.

The Cy probe utilizes flanking centromeric and telomeric probes for each gene, providing partial coverage of *RARA* and no direct coverage of *PML*. In contrast, the Abbott probe spans a broader region, covering most of the *PML* (15q22–24) and *RARA* (17q21) loci, including substantial flanking sequences (Figure 1). All eight APL cases identified were processed with the Cy-dual-color dual-fusion probe. The two cases that were further evaluated were also processed using the Ab probe set. The 2024 International System for Human Cytogenetic Nomenclature was used for reporting karyotype and FISH results.

Genomic DNA extracted from the collected bone marrow samples was processed using the QIAseq Targeted DNA Human Myeloid Neoplasms Panel (QIAGEN GmbH, Hilden, Germany), involving the 141 myeloid-related genes [6]. Detected variant calls were classified according to the American College of Medical Genetics and Genomics (ACMG) and American Molecular Pathology guidelines using QIAGEN Clinical Insight–Interpret software, version 6.0.20200522 [7].

## 3. Results

The conventional cytogenetic and FISH results using the *PML::RARA* probe(s) are presented in Table 1.

### 3.1. Case 1

**Figure 2:**
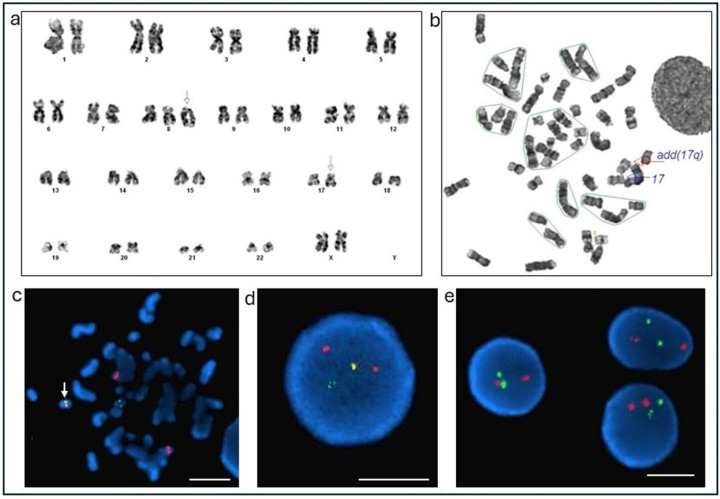
Case 1: Conventional Cytogenetics and FISH results. (**a**) Karyotype: 47,XX,+der(8)add(8)(p11.2)dup(8)(q13q22),add(17)(q21)[cp9]/46,XX[11]. Karyogram shows the abnormal metaphase findings. The arrow points to the abnormal chromosomes 8 and 17. (**b**) Metaphase with abnormal 17q [add(17q)]. FISH results using *PML* (red/orange) and *RARA* (green) probes from Ab and Cy (**c**) Metaphase FISH show *PML::RARA* fusion signal (White arrow, with fused orange and green signal) on the abnormal chromosome 17q (White arrow) using Ab probe. (**d**) *PML::RARA* FISH on interphase cells using Ab probe set shows an atypical single *PML::RARA* fusion, two *PML,* and one *RARA* signal pattern. (**e**) *PML::RARA* FISH on interphases using a Cy probe set shows normal two *PML* and two *RARA* signal patterns. Scale bar (white) = 5 μm.

The first case of interest, received in the cytogenetics laboratory in October 2020, involved a bone marrow specimen from a 29-year-old female who presented with persistent fevers and malaise and tested positive for COVID-19. She continued to have fevers despite taking acetaminophen and ibuprofen at high doses. She was subsequently admitted to the emergency department with anemia, thrombocytopenia, and leukocytosis. On Day 1, laboratory evaluation at an outside institution revealed a white blood cell count of 26,000/μL and circulating blasts on peripheral smear, raising clinical suspicion for APL. All-trans retinoic acid (ATRA) 40 mg twice daily was given pending an APL diagnosis. Diagnostic bone marrow aspirate revealed a hypercellular marrow (100%) with 92% blast equivalents, including atypical hypogranular promyelocytes and markedly decreased trilineage hematopoiesis. Morphologic evaluation showed blasts with a spectrum of appearances, including folded cerebriform nuclei and a subset with bilobed nuclei typical of microgranular APL. Occasional Auer rods were identified. Empiric treatment with prednisone and tretinoin was initiated. Due to worsening leukocytosis, a bone marrow biopsy was performed on Day 4, and the morphologic assessment was concerning for APL. The patient care was transferred to the current institution for further treatment on Day 8. Peripheral blood flow cytometry demonstrated that atypical myeloid precursors accounted for 93% of total events. These cells expressed CD11b, CD13, CD15 (dim), CD33, CD38, CD64, CD117 (dim), CD123, and cytoplasmic MPO, while lacking expression of CD11c, CD14, CD34, HLA-DR, and all lymphoid markers. NGS detected four variants in the diagnostic sample: a tier 1A *FLT3* p.D835V variant and three tier 3 *KDM6A* p.R1111P, *RELN* p.T3242M, and *RELN* p.D293N variants. Follow-up NGS performed 1 month after the initial test detected a single *RELN* p.D293N variant. Conventional cytogenetics yielded 47,XX,+der(8)add(8)(p11.2)dup(8)(q13q22),add(17)(q21)[cp9]/46,XX[11] karyotype (Figure 2a).

Initial FISH analysis using the Cy dual-color, dual-fusion probe for *PML::RARA* rearrangement was negative (Figure 2e). Conventional karyotyping revealed additional genetic material at the 17q21 breakpoint (Figure 2a,b). The patient was started on hydroxyurea on Day 4 for hyperleukocytosis and developed spontaneous tumor lysis syndrome and DIC. She received allopurinol. Fresh-frozen plasma was administered to manage low fibrinogen levels.

Given the high clinical suspicion for APL, molecular analysis for *PML::RARA* mRNA was performed, which confirmed a fusion involving *RARA* and the breakpoint cluster region 3 (bcr3, intron 3) of the *PML* gene. Due to the limited genomic coverage of the Cytocell probe, FISH was repeated using the Ab probe, which targets a broader region of the *PML* and *RARA* loci (Figure 1). The Ab probe yielded a positive result, demonstrating an atypical *PML::RARA* fusion (Figure 2d). Metaphase FISH confirmed a cryptic fusion signal on the abnormal chromosome 17q (Figure 2c).

The patient initially received four days of induction chemotherapy with a 7+3 regimen beginning on Day 9 and was subsequently transitioned to a high-risk APL treatment protocol with ATRA and arsenic trioxide (AO), initiated on Day 12. Prophylaxis for differentiation syndrome was completed on Day 20. The patient remains in remission (last assessed March 2025).

### 3.2. Case 2

The second case involved a 30-year-old female who presented to an outside hospital emergency department in December 2021 (Day 1), with left wrist erythema, swelling, and a fever of 100.7 °F. Laboratory evaluation revealed pancytopenia, prompting a bone marrow biopsy that was suspicious for APL. Outside bone marrow evaluation reported hypercellular marrow with decreased trilineage hematopoiesis and 77% blasts and promyelocytes. She was subsequently transferred to the University of Kansas Health System, where a repeat bone marrow biopsy was performed on Day 2. Morphologic evaluation of the bone marrow revealed a hypercellular marrow (~90%) with markedly decreased trilineage hematopoiesis and 77% blasts, including atypical promyelocytes with abundant cytoplasmic granules and occasional Auer rods. Flow cytometry findings were consistent with APL. The NGS study detected no clinically actionable or oncogenic gene variants. A *KLHL6* p.Y129N variant of unknown significance was detected at an allelic fraction of 49.0%. The functional consequences and clinical significance of this variant are not established.

**Figure 3:**
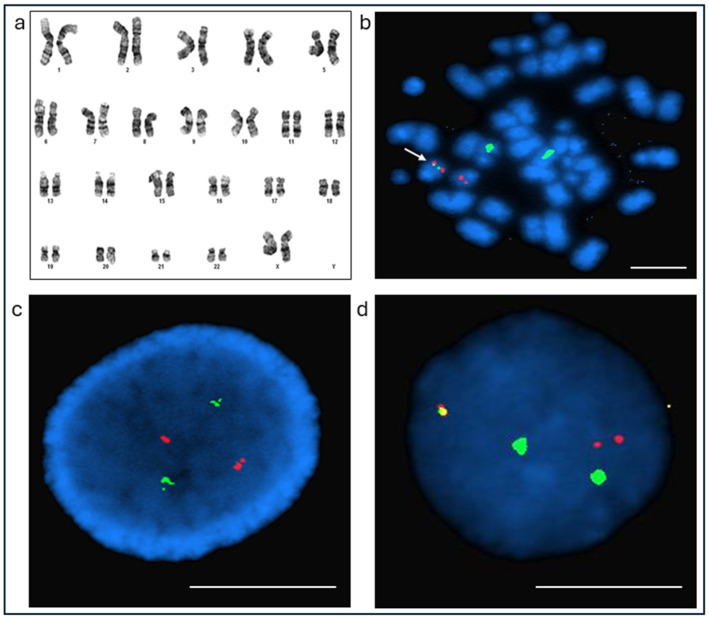
Case 2: Conventional Cytogenetics and FISH results using *PML* (red/orange) and *RARA* (green) probes from Ab and Cy. (**a**) Karyotype: A normal female karyotype, 46,XX[20]. (**b**) Metaphase FISH using Cy probe set show a cryptic *PML::RARA* fusion signal on chromosome 15 (White arrow, with fused red and green signal). (**c**) *PML::RARA* FISH on interphases using Ab probe set shows normal two *PML* and two *RARA* signals. (**d**) *PML::RARA* FISH on interphase nuclei using a Cy probe set shows an atypical single *PML::RARA* fusion, one *PML*, and two *RARA* signals. Scale bar (white) = 5 μm.

Treatment with ATRA and arsenic trioxide was initiated the same day. Cytogenetic analysis revealed a normal karyotype (Figure 3a), and initial FISH testing with the Ab probe for the *PML::RARA* rearrangement was negative (Figure 3c). Given the strong clinical suspicion for APL, additional molecular studies were pursued. qRT-PCR confirmed the presence of the *PML::RARA* fusion transcript. FISH analysis was repeated using the Cy probe, which yielded a positive result with an atypical signal pattern (Figure 3d). Metaphase FISH using the Cy probe confirmed a cryptic *PML::RARA* fusion on chromosome 15, consistent with a cryptic *RARA* insertion event (Figure 3b). The patient was discharged on January 13 with close outpatient follow-up through the University of Kansas Health System Hematology service for continued management of ATRA/arsenic therapy and monitoring of neutropenia. No further bone marrow biopsies were performed, and yearly complete blood count (CBC) evaluations remained unremarkable. For non-hematologic concerns with autism with cognitive delay, bipolar disorder, seizures, and prior cardiac problems, the patient is followed up by the general medicine and psychiatric teams. The patient was last followed in December 2025, with an unremarkable CBC. The peripheral blood test for quantitative evaluation of the *PML::RARA* transcripts is pending at the time of manuscript preparation.

## 4. Discussion

The diagnosis of APL is typically confirmed by detection of the reciprocal translocation t(15;17)(q24;q21), which results in the *PML::RARA* gene fusion. Conventional cytogenetic techniques, such as karyotyping and FISH, are commonly used to identify this rearrangement. However, in rare cases, the characteristic translocation may be absent due to cryptic rearrangements, including submicroscopic insertions of *RARA* into *PML* or other complex structural variants. These cryptic forms of APL may present with variable cytogenetic findings or even a normal karyotype, and may yield negative FISH results, increasing the risk of misdiagnosis and delayed treatment.

The *PML* and *RARA* FISH probe coverage differs significantly between Ab and Cy. The Ab probe is larger and spans both the *PML* and *RARA* genes, whereas the Cy probes primarily target the flanking regions of these genes, covering only a small portion of 3′ *RARA*. As shown in Figure 1, the Cy probes are shorter than the Ab probes. In a typical 15;17 translocation, the derivative chromosome 15 is pathognomonic and leads to expression of the *PML*::*RARA* fusion transcript [8]. In Case 1, molecular RT-PCR detected a BCR3-*PML*::*RARA* fusion transcript, while FISH using an Ab probe revealed a cryptic *PML* insertion into the *RARA* region on chromosome 17 and Cy was normal. Because the Ab probe spans the entire *PML* gene, it offers a broader advantage over the Cy probe set for detecting cryptic *PML* insertion events that are missed by Cy probes, which do not cover the PML gene but its flanking regions (Figure 1). Molecular qRT-PCR confirmed the presence of the *PML*::*RARA* fusion transcript in Case 2, the medical records lacked details regarding the isoform in Case 2. A normal karyotype in this case did not help to further investigate the discrepancy in results between the Ab and Cy *PML::RARA* probes. The two cases presented in this study are limited by the absence of direct sequencing of the *PML* and *RARA* genes due to insufficient diagnostic specimens. This would have enabled precise breakpoint mapping at the *PML* and *RARA* loci on chromosomes 15 and 17, respectively, and provided better insight into the limitations of the Ab and Cy probe sets.

Several studies have described cryptic and atypical *PML::RARA* gene fusions, occurring with or without a normal conventional karyotype, primarily detected using the Abbott dual-color dual-fusion FISH probe set [9,10,11]. In one such study, FISH analysis revealed a submicroscopic insertion of chromosome 15 material into 17q, despite a normal karyotype [10]. While FISH-negative cases were infrequent, these studies did not evaluate alternative FISH probe sets from other vendors, which may differ in genomic coverage and sensitivity. As a result, the limitation of these alternative probe sets for detecting *PML::RARA* rearrangements remains unaddressed. Consequently, the findings from these prior studies cannot be directly correlated with the cases presented in the current study.

In APL, co-occurring mutations are present in 70% of the cases [8,12]. *FLT3*-ITD and *FLT3*-D835 are the most common gene mutations seen in both pediatric and adult populations. Other non-recurring mutations involve genes in the MAPK pathway and transcription regulatory genes, including *KDM6A* [8,13,14]. However, there is limited evidence of co-occurring mutations in the literature with a variant or atypical *PML::RARA* gene rearrangement [8,15,16,17]. A synergistic antileukemogenic effect of ATRA and AO therapy was observed in APL harboring the *FLT3* mutation [18,19]. This observation is supported by the current finding in case 1, in which, after post-ATRA and AO therapy, NGS of the follow-up specimen showed loss of the *FLT3* oncogenic variant.

Advanced cytogenetic techniques offer a valuable alternative for investigating atypical insertional translocations. Optical genome mapping (OGM), an emerging and cutting-edge cytogenomic molecular technology, enables genome-wide detection of structural rearrangements at gene-level resolution. Multiple studies have demonstrated its utility in identifying submicroscopic structural abnormalities that are often missed by conventional karyotyping. Furthermore, OGM has proven effective in elucidating complex chromosomal rearrangements, including cryptic fusion genes, thereby enhancing diagnostic precision and genomic characterization [20,21]. OGM has identified a similar insertion event involving the *PML*: *RARA* fusion insertion into the *RARA* gene on 17q in a normal karyotype, and an atypical *PML::RARA* FISH signal pattern was detected using the Ab *PML::RARA* dual color dual fusion FISH probe [22].

The two cases presented here illustrate that neither the Ab nor the Cy FISH probes alone are sufficient to detect all possible rare *PML::RARA* fusion variants. However, using both probe sets may improve detection sensitivity. A study by Campbell et al. [23] evaluated ten cases of APL with *RARA* insertions into *PML* using both Ab and Cy dual-fusion FISH probes. The Cy probe detected the fusion in 7 of 10 cases, while the Ab probe yielded equivocal results in 3 cases and was negative in the remaining 7 cases. Notably, our first case demonstrated the opposite outcome: the Ab probe was positive while the Cy probe was negative. This case involved a *PML* insertion into *RARA*, whereas the Campbell study focused exclusively on *RARA* insertions into *PML*. These findings suggest that reflex testing with an alternate FISH probe is warranted in rare cases with high clinical suspicion for APL and negative initial FISH results. In both cases, ATRA therapy was initiated based on a strong clinical suspicion of APL, before diagnostic confirmation, to prevent DIC. FISH studies did not delay therapy initiation in either case. However, subsequent diagnostic confirmation was crucial for continuing ATRA therapy and avoiding unnecessary chemotherapy [24,25].

The proposed FISH testing algorithm for APL diagnosis should begin with dual-color, dual-fusion FISH for *PML::RARA* using a commercially available probe validated in a clinical laboratory in accordance with regulatory guidelines. A positive result confirms APL. If FISH is negative but morphology and/or immunophenotype strongly suggest APL, reflex testing should include an alternative vendor’s FISH probe with different genomic coverage and RT-PCR for *PML::RARA* transcript detection to capture rare cryptic rearrangements. If the second FISH probe is negative but RT-PCR is positive, direct sequencing or OGM should be considered to identify rare RARA fusions and determine precise breakpoints in *PML* and *RARA* genes.

## 5. Conclusions

Cryptic *PML::RARA* rearrangements in APL can evade detection by standard FISH probes due to limitations in probe design and genomic coverage. FISH remains the essential diagnostic assay for detecting the *PML::RARA* rearrangement, given its short TAT. These cases underscore the need for reflex testing with alternative probe sets in patients with an initial negative FISH result and for further follow-up with molecular confirmation via qRT-PCR or OGM in patients with high clinical suspicion for APL. This approach helps prevent false-negative FISH results, reducing diagnostic delays and enabling timely initiation of life-saving therapy.

## Figures and Tables

**Table 1:** Eight APL cases with details of the *PML::RARA* FISH probes used, along with cytogenetic findings.

APL Case	Cytogenetics Result	FISH Probe
1	Karyotype: 46,XX,t(15;17)(q24;q21)[20] FISH: nuc ish (PML,RARA)x3(PML con RARA)x2[189/200]	Cy
2	Karyotype: 46,XY,t(15;22;17)(q24;q13;q21)[18]/46,XY [2] FISH: nuc ish (PMLx2,RARAx3)(PML con RARA)x1[176/200]	Cy
3	Karyotype: 46,XX,t(15;17)(q24;q21)[20] FISH: nuc ish (PML,RARA)x3(PML con RARA)x2[196/200]|	Cy
4	FISH: nuc ish (PMLx3,RARAx2)(PML con RARA)x1[27/500]	Cy
5	Karyotype: 46,XX,t(15;17)(q24;q21)[10]/46,XX[6] FISH: nuc ish (PML,RARA)x3(PML con RARA)x2[140/200]	Cy
6	Karyotype: 46,XX,t(15;17)(q24;q21)[15]/46,XX[5] nuc ish (PML,RARA)x3(PML con RARA)x2[165/200]	Cy
7 (Case 1)	Karyotype: 47,XX,+der(8)add(8)(p11.2)dup(8)(q13q22),add(17)(q21)[cp9]/46,XX[11] FISH	
	FISH: nuc ish (PML,RARA)x2[200]	Cy
	FISH: nuc ish (PMLx3,RARAx2)(PML con RARA)x1[160/200]	Ab
8 (Case 2)	Karyotype: 46,XX[20]	
	FISH: nuc ish (PMLx2,RARAx3)(PML con RARA)x1[150/200]	Cy
	FISH: nuc ish (PML,RARA)x2[200]	Ab

Note: Numbers in the square brackets under Cytogenetics result indicate the number of metaphases or interphase nuclei cells.

## Data Availability

The raw data supporting the conclusions of this article will be made available by the authors on request.

## Abbreviations

The following abbreviations are used in this manuscript:

APL	Acute Promyelocytic Leukemia
DIC	Disseminated Intravascular Coagulation
*PML*	Promyelocytic Leukemia Gene
*RARA*	Retinoic Acid Receptor Alpha Gene
FISH	Fluorescence In Situ Hybridization
qRT-PCR	Quantitative Real-Time Polymerase Chain Reaction
NGS	Next-generation sequencing
TAT	Turnaround Time
Ab	Abbott Molecular
Cy	CytoCell
ATRA	All-Trans Retinoic Acid
AO	Arsenic trioxide
